# Supplementary material for: High-throughput peptide-centric local stability assay extends protein–ligand identification to membrane proteins, tissues and bacteria
Source: Nat Struct Mol Biol. 2025 Nov 5;33(1):184–92. doi: 10.1038/s41594-025-01699-y (PMC12819154; doi:10.1038/s41594-025-01699-y)
Supplement: Supplementary file 1 — Reporting Summary [file 41594_2025_1699_MOESM1_ESM.pdf]

nature portfolio

Corresponding author(s): Mikihail M Savitski

Last updated by author(s): Sep 22, 2025

Reporting Summary

Nature Portfolio wishes to improve the reproducibility of the work that we publish. This form provides structure for consistency and transparency in reporting. For further information on Nature Portfolio policies, see our Editorial Policies and the Editorial Policy Checklist.

Statistics

For all statistical analyses, confirm that the following items are present in the figure legend, table legend, main text, or Methods section.

n/a	Confirmed	
☐	☒	The exact sample size (*n*) for each experimental group/condition, given as a discrete number and unit of measurement
☐	☒	A statement on whether measurements were taken from distinct samples or whether the same sample was measured repeatedly
☐	☒	The statistical test(s) used AND whether they are one- or two-sided<br>*Only common tests should be described solely by name; describe more complex techniques in the Methods section.*
☒	☐	A description of all covariates tested
☐	☒	A description of any assumptions or corrections, such as tests of normality and adjustment for multiple comparisons
☐	☒	A full description of the statistical parameters including central tendency (e.g. means) or other basic estimates (e.g. regression coefficient) AND variation (e.g. standard deviation) or associated estimates of uncertainty (e.g. confidence intervals)
☐	☒	For null hypothesis testing, the test statistic (e.g. *F*, *t*, *r*) with confidence intervals, effect sizes, degrees of freedom and *P* value noted<br>*Give P values as exact values whenever suitable.*
☒	☐	For Bayesian analysis, information on the choice of priors and Markov chain Monte Carlo settings
☒	☐	For hierarchical and complex designs, identification of the appropriate level for tests and full reporting of outcomes
☐	☒	Estimates of effect sizes (e.g. Cohen's *d*, Pearson's *r*), indicating how they were calculated

Our web collection on statistics for biologists contains articles on many of the points above.

Software and code

Policy information about availability of computer code

Data collection	The mass spectrometers Orbitrap Exploris 480 and Astral were used for data acquisition.
Data analysis	Raw data was processed with DIA-NN (version 1.8.1). Protein structures are visualized with PyMOL (version 2.5.8). Data analysis was performed with R (4.3.3). Gene ontology analysis is performed with clusterProfiler (4.10.1). The code to reproduce the analysis and the figures can be found at Github (https://github.com/nicohuttmann/HT-PELSA).

For manuscripts utilizing custom algorithms or software that are central to the research but not yet described in published literature, software must be made available to editors and reviewers. We strongly encourage code deposition in a community repository (e.g. GitHub). See the Nature Portfolio guidelines for submitting code & software for further information.

Data

Policy information about availability of data

All manuscripts must include a data availability statement. This statement should provide the following information, where applicable:

- Accession codes, unique identifiers, or web links for publicly available datasets
- A description of any restrictions on data availability
- For clinical datasets or third party data, please ensure that the statement adheres to our policy

The raw mass spectrometry proteomics data, Fasta files, and DIA-NN output results have been deposited to the ProteomeXchange Consortium through the PRIDE partner repository with the dataset identifier PXD062869. Supplementary Data are available via Figshare at https://doi.org/10.6084/m9.figshare.30191833

Source Data for each figure and Supplementary datasets have been provided along with the paper. Protein structures are downloaded from Protein Data Bank or Alphafold.

Research involving human participants, their data, or biological material

Policy information about studies with human participants or human data. See also policy information about sex, gender (identity/presentation), and sexual orientation and race, ethnicity and racism.

Reporting on sex and gender	*Use the terms sex (biological attribute) and gender (shaped by social and cultural circumstances) carefully in order to avoid confusing both terms. Indicate if findings apply to only one sex or gender; describe whether sex and gender were considered in study design; whether sex and/or gender was determined based on self-reporting or assigned and methods used. Provide in the source data disaggregated sex and gender data, where this information has been collected, and if consent has been obtained for sharing of individual-level data; provide overall numbers in this Reporting Summary. Please state if this information has not been collected. Report sex- and gender-based analyses where performed, justify reasons for lack of sex- and gender-based analysis.*
Reporting on race, ethnicity, or other socially relevant groupings	*Please specify the socially constructed or socially relevant categorization variable(s) used in your manuscript and explain why they were used. Please note that such variables should not be used as proxies for other socially constructed/relevant variables (for example, race or ethnicity should not be used as a proxy for socioeconomic status). Provide clear definitions of the relevant terms used, how they were provided (by the participants/respondents, the researchers, or third parties), and the method(s) used to classify people into the different categories (e.g. self-report, census or administrative data, social media data, etc.) Please provide details about how you controlled for confounding variables in your analyses.*
Population characteristics	*Describe the covariate-relevant population characteristics of the human research participants (e.g. age, genotypic information, past and current diagnosis and treatment categories). If you filled out the behavioural & social sciences study design questions and have nothing to add here, write "See above."*
Recruitment	*Describe how participants were recruited. Outline any potential self-selection bias or other biases that may be present and how these are likely to impact results.*
Ethics oversight	*Identify the organization(s) that approved the study protocol.*

Note that full information on the approval of the study protocol must also be provided in the manuscript.

Field-specific reporting

Please select the one below that is the best fit for your research. If you are not sure, read the appropriate sections before making your selection.

☒ Life sciences ☐ Behavioural & social sciences ☐ Ecological, evolutionary & environmental sciences

For a reference copy of the document with all sections, see nature.com/documents/nr-reporting-summary-flat.pdf

Life sciences study design

All studies must disclose on these points even when the disclosure is negative.

Sample size	No statistical methods were used to predetermine sample size. To determine the significantly changed peptides or proteins, each experiment is performed with four replicates. Four replicates are sufficient to perform empirical Bayes moderated t-statistics to get the p values. As showed in the manuscript, after statistics analysis, the proteins with smallest p values are always known targets, confirming the effectiveness of using four replicates. The dose-response experiments were also performed with four replicates, and we required the target peptides fit the dose-response curves in at least three replicates; this allowed us to calculate the coefficient of variation (CV) of the pEC50 values derived from different replicates. By evaluating the CV and applying a cutoff, we were able to obtain consistent and reliable pEC50 values for each target peptide.
Data exclusions	For single concentration HT-PELSA experiment involving 8 samples, peptides with missing values in any of the replicates are removed. Since the number of the missing values increases with the number of sample, in dose-response HT-PELSA experiments involving 32 samples, we require peptides be quantified in at least three replicates and peptides quantified in less than three replicates, are removed.
Replication	Conclusions were drawn from reproducible effects in all replicates of the datasets. All experiments are performed using four replicates.
Randomization	The protein samples used for different treatments are from the same cell or tissue lysates. The lysates were thoroughly mixed and evenly distributed into separate tubes for vehicle or ligand treatment, ensuring no bias during distribution.
Blinding	This is not relevant to the study. After incubation with ligand or vehicle, the lysates are distributed into a 96-well plate; from that point onward, all samples are processed under identical conditions and for the same duration within the 96-well plate.

Reporting for specific materials, systems and methods

We require information from authors about some types of materials, experimental systems and methods used in many studies. Here, indicate whether each material, system or method listed is relevant to your study. If you are not sure if a list item applies to your research, read the appropriate section before selecting a response.

Materials & experimental systems

n/a	Involved in the study	
☒	☐	Antibodies
☐	☒	Eukaryotic cell lines
☒	☐	Palaeontology and archaeology
☐	☒	Animals and other organisms
☒	☐	Clinical data
☒	☐	Dual use research of concern
☒	☐	Plants

Methods

n/a	Involved in the study	
☒	☐	ChIP-seq
☒	☐	Flow cytometry
☒	☐	MRI-based neuroimaging

Eukaryotic cell lines

Policy information about cell lines and Sex and Gender in Research

Cell line source(s)	K562 cells are acquired from American Type Culture Collection (ATCC, CCL-243).
Authentication	Cells were authenticated by STR profiling by vendors.
Mycoplasma contamination	No mycoplasma contamination was discovered during the cell culture.
Commonly misidentified lines (See ICLAC register)	No misidentified cell lines have been used in this study.

Animals and other research organisms

Policy information about studies involving animals; ARRIVE guidelines recommended for reporting animal research, and Sex and Gender in Research

Laboratory animals	The liver sample was collected from a 2-month-old C57BL/6 male mouse, and the heart sample from a 4-month-old C57BL/6J male mouse.
Wild animals	The study did not involve wild animals.
Reporting on sex	Sex-based analysis was not performed, as we believe that sex may not significantly impact the proteome of non-reproductive organs, such as the liver and heart.
Field-collected samples	The study did not involve sample collected from the field.
Ethics oversight	All animal care and procedures were conducted in line with EMBL regulations and guidelines for the use of animals in research and were reviewed and approved by the Institutional Animal Care and Use Committee (IACUC). All mouse experiments were performed using approved protocols by the European Molecular Biology Laboratory (EMBL) ethics committee (license 21-002_HD_MZ).

Note that full information on the approval of the study protocol must also be provided in the manuscript.

Plants

Seed stocks	*Report on the source of all seed stocks or other plant material used. If applicable, state the seed stock centre and catalogue number. If plant specimens were collected from the field, describe the collection location, date and sampling procedures.*
Novel plant genotypes	*Describe the methods by which all novel plant genotypes were produced. This includes those generated by transgenic approaches, gene editing, chemical/radiation-based mutagenesis and hybridization. For transgenic lines, describe the transformation method, the number of independent lines analyzed and the generation upon which experiments were performed. For gene-edited lines, describe the editor used, the endogenous sequence targeted for editing, the targeting guide RNA sequence (if applicable) and how the editor was applied.*
Authentication	*Describe any authentication procedures for each seed stock used or novel genotype generated. Describe any experiments used to assess the effect of a mutation and, where applicable, how potential secondary effects (e.g. second site T-DNA insertions, mosiacism, off-target gene editing) were examined.*
